# The Role of Tissue-Resident Macrophages in the Development and Treatment of Inflammatory Bowel Disease

**DOI:** 10.3389/fcell.2022.896591

**Published:** 2022-05-26

**Authors:** Shengjie Ma, Jiaxin Zhang, Heshi Liu, Shuang Li, Quan Wang

**Affiliations:** Department of Gastrointestinal Surgery, The First Hospital of Jilin University, Chang Chun, China

**Keywords:** inflammatory bowel disease, ulcerative colitis, Crohn’s disease, tissue-resident macrophage, macrophage polarization

## Abstract

Inflammatory bowel disease (IBD), comprising Crohn’s disease and ulcerative colitis, is a refractory disease with many immune abnormalities and pathologies in the gastrointestinal tract. Because macrophages can distinguish innocuous antigens from potential pathogens to maintain mucosa barrier functions, they are essential cells in the intestinal immune system. With numerous numbers in the intestinal tract, tissue-resident macrophages have a signiﬁcant effect on the constant regeneration of intestinal epithelial cells and maintaining the immune homeostasis of the intestinal mucosa. They also have a significant influence on IBD through regulating pro-(M1) or anti-inﬂammatory (M2) phenotype polarization according to different environmental cues. The disequilibrium of the phenotypes and functions of macrophages, disturbed by intracellular or extracellular stimuli, influences the progression of disease. Further investigation of macrophages’ role in the progression of IBD will facilitate deciphering the pathogenesis of disease and exploring novel targets to develop novel medications. In this review, we shed light on the origin and maintenance of intestinal macrophages, as well as the role of macrophages in the occurrence and development of IBD. In addition, we summarize the interaction between gut microbiota and intestinal macrophages, and the role of the macrophage-derived exosome. Furthermore, we discuss the molecular and cellular mechanisms participating in the polarization and functions of gut macrophages, the potential targeted strategies, and current clinical trials for IBD.

## 1 Introduction

In recent years, the morbidity of inflammatory bowel disease (IBD) has increased globally, especially in Africa and Asia, and is spreading worldwide with accelerated speed (Windsor and Kaplan, 2019). IBD is an idiopathic, refractory and chronic disease, with clinical symptoms including abdominal pain, rectal bleeding, weight loss, diarrhea, and anemia, that requires life-long medication (Roda et al., 2020; Seyed Tabib et al., 2020). Conventionally, IBD is mainly divided into Crohn’s disease (CD) and ulcerative colitis (UC), along with different locations and inflammatory types, respectively (Torres et al., 2017; Ungaro et al., 2017). CD affects almost every part of the gastrointestinal tract and tissue layers, and the most frequent locations are the terminal ileum and colon (Torres et al., 2017; Roda et al., 2020). However, UC mainly involves the superficial intestinal mucosa of the colon, including the proximal end of the rectum (Ungaro et al., 2017; Kobayashi et al., 2020). Approximately 5 million people worldwide are affected with IBD, yet the etiology of IBD is not known, and there are no cures so far (Kaplan, 2015). The continuation of this trend is likely to increase the worldwide population of patients affected by IBD to tens of millions or more in the next few decades (Ananthakrishnan, 2015). More importantly, recent discoveries revealed that patients with IBD are more susceptible to colorectal cancer (Nadeem et al., 2020). Thus, the prevention and treatment of IBD are extremely necessary and important.

The etiology of IBD arises from intricate interactions between the immune system, genetic predisposition, environmental cues, and the gut microbiome (Kobayashi et al., 2020; Roda et al., 2020). Under ideal conditions, intestinal inflammation is a self-limiting process that combats harmful pathogens and rapidly returns to homeostasis (Schett and Neurath, 2018). However, a disequilibrium between the mucosal immune system and the commensal ecosystem occurs in the exacerbation of IBD, because of the imbalance of the immunological mechanisms underlying the resolution of inflammation (Hunter, 2012; Schett and Neurath, 2018; Graham and Xavier, 2020). Intestinal macrophages are an integral part of the normal intestinal tissues that can engulf microorganisms and present antigens to activate T cells, as well as prevent excessive inflammation through specific molecular and cellular mechanisms (Smythies et al., 2005; Garrett et al., 2010; Mowat, 2018). According to the microenvironmental cues, macrophages could polarize into two different phenotypes: the classically activated (M1) or alternatively activated (M2) macrophages (Funes et al., 2018; Locati et al., 2020). When the intestinal barrier function is impaired, the efferocytosis effect leads to macrophages skewing toward M2 subtypes functionally, producing cytokines, chemokines and lipid mediators that participate in healing the intestinal mucosal barrier and maintaining homeostasis (Elliott et al., 2017; Na et al., 2019). Various cytokines and other soluble factors, including prostaglandin E2 (PGE2), bone morphogenetic protein 2 (BMP2), and WNT ligands, can be produced by gut macrophages that stimulate the expansion of epithelial cells and participate in the enteric nervous system or intestinal mucosal barrier to help maintain tissue homeostasis (Bain and Mowat, 2014). Meanwhile, intestinal macrophages maintain tolerance *via* promoting the proliferation of antigen-specific CD4^+^CD25^+^ regulatory T (T-reg) cells primarily by producing interleukin (IL)-10 in order to inhibit unrestrained inflammation as a response to innocuous commensal microbes (O'Garra et al., 2004; Hedrich and Bream, 2010).

In addition, the lack of response in patients with IBD to anti-tumor necrosis factor (TNF) therapy was found to be related to a defect in the IL-10 signaling pathway, which is crucial for biologics-induced macrophages skewing toward M2 subsets (Vos et al., 2012; Roda et al., 2016; Koelink et al., 2020). Therefore, intestinal macrophages play major roles in the establishment and maintenance of intestinal homeostasis. As a consequence, disequilibrium of intestinal macrophages could lead to a loss of tolerance to harmless microbes and food antigens, which is considered to underlie the excessive inflammation in IBD (de Souza and Fiocchi, 2016). In view of these findings, it is important to analyze the potential contribution of macrophages to intestinal chronic inflammation, which may offer us novel insights into the pathogenesis and therapeutic strategies in IBD.

In this review, we outline the characteristics of the origin and maintenance of macrophages, as well as their interaction with other immune cells. Then, we delineate the cellular and molecular mechanisms that underlie the differentiation and function of intestinal macrophages. Furthermore, we describe the interaction between gut microbiota and intestinal macrophages, and the role of the macrophage-derived exosome. Finally, potential treatment targets, applicable drugs and current clinical trials for IBD are discussed.

## 2 Origin and Maintenance of Intestinal Macrophages

The gastrointestinal tract harbors copious amounts of tissue macrophages, orchestrating innate and adaptive immune systems, helping to maintain homeostasis *via* tolerance to commensal microbes, as well as resisting potentially harmful pathogens (Yip et al., 2021). In the past, the origin of tissue macrophages was considered to be the blood monocytes, deriving from precursors in the bone marrow (van Furth et al., 1972; Bain and Mowat, 2014). With the development of novel techniques, there is mounting evidence showing that monocytes are not the exclusive origin of tissue-resident macrophages (Wu and Hirschi, 2020). Macrophages arise in two different ways: they can be differentiated from blood-derived monocytes and embryo-derived precursors, deriving from the yolk sac and/or fetal liver, established in the tissue before birth (Shaw et al., 2018; Wu and Hirschi, 2020). Intestinal tissue-resident macrophages, derived from embryonic precursors, maintain themselves *via* self-renewal *in situ* throughout adulthood but also require constant replenishment by circulating monocytes in the blood (Shaw et al., 2018). This is in contrast to macrophages in other tissues, such as microglia in the central nervous system and Langerhans cells in the liver, that maintain themselves independently and self-renew *in situ* throughout adult life (Yona et al., 2013; Ginhoux and Guilliams, 2016).

With progress in recent research, traditional understanding has been challenged, and embryo-derived macrophages were found to sustain themselves in the adult intestine (Shaw et al., 2018). In adult mice, parts of intestinal macrophages, characterized by high expression levels of CD4 and apoptotic cell-uptake receptor Tim-4 (Tim-4^+^CD4^+^ macrophages), were demonstrated to be long-lived and locally self-renewed independent of monocytes, while Tim-4^−^CD4^+^ macrophages were found to have a slow turnover from blood monocytes (Shaw et al., 2018). In contrast, Tim-4^−^CD4^−^ macrophages were short-lived and relied on continuous replenishment of blood monocytes (Shaw et al., 2018). Similar results have been found in duodenal transplant patients’ mucosa, along with short-lived monocyte-derived macrophage subsets, different and dominant subsets with a slower replenishment rate were confirmed (Bujko et al., 2018). Similarly, another study in mice found that self-renewal embryo-derived macrophages and bone marrow-derived monocytes concomitantly reside in the lamina propria and muscularis externa layer of the intestine, persisting throughout adulthood (De Schepper et al., 2018). Depletion of self-maintaining macrophages could lead to a series of problems, such as injury of the submucosal vascular network, damage of enteric neurons, impaired neuro-evoked gastrointestinal section, and weakened intestinal motility (De Schepper et al., 2018). As noted above, parabiosis experiments (Guilliams et al., 2013; Hashimoto et al., 2013; Jakubzick et al., 2013; Epelman et al., 2014) and genetic fate-mapping experiments (Ginhoux et al., 2010; Hoeffel et al., 2012; Schulz et al., 2012; Yona et al., 2013) have proved that macrophages in the adult gut originating from yolk-sac precursors maintain self-renewal throughout life, thus existing independently of bone-marrow precursors for long-lasting maintenance. Given that there are different intestinal macrophage subsets with strong heterogeneity and distinct functional characteristics, future studies on macrophages in IBD should be focusing on altering the inflammatory phenotype, in parallel with keeping the protective tissue-resident subpopulations intact.

**FIGURE 1:**
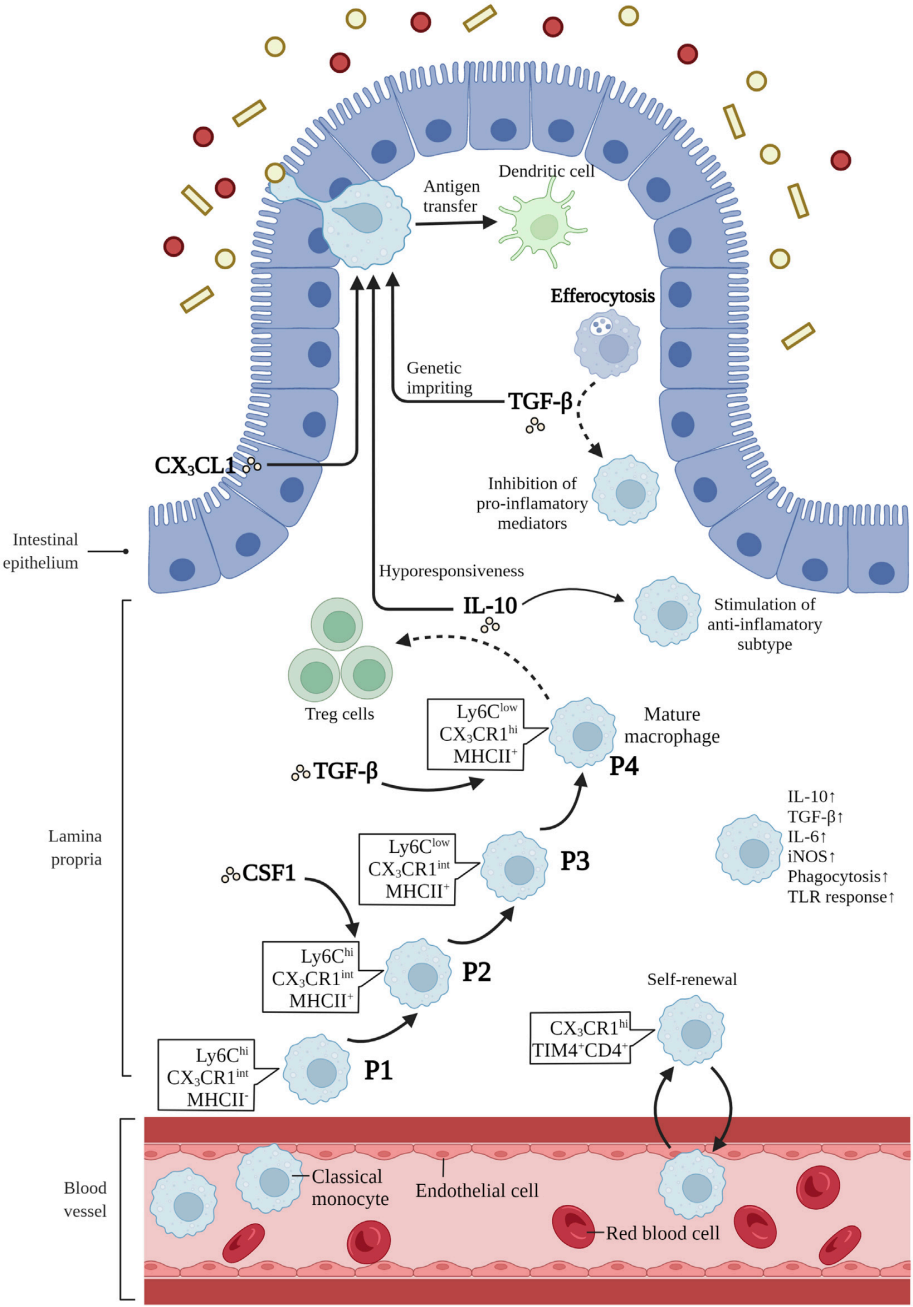
Differentiation and function of intestinal macrophages. Intestinal macrophages are replenished by blood-derived monocytes through a series of differential intermediates in a way of “monocyte waterfall”. lymphocyte antigen 6C-high (Ly6C^hi^) CX3C-chemokine receptor 1-intermediate (CX_3_CR1^int^) major histocompatibility complex II (MHCII^−^) (P1) monocytes enter the lamina propria, acquiring the expression of MHCII (P2), and then downregulating Ly6C expression (P3). Finally, these cells upregulate the expression of CX_3_CR1, CD64, and F4/80, giving rise to mature macrophages (P4). T cell immunoglobulin mucin receptor 4-positive (Tim-4^+^) CD4^+^ macrophages have been demonstrated to be long-lived and locally self-renewed independent of monocytes. Macrophage colony-stimulating factor 1 (CSF1) is a critical cytokine in the survival, proliferation and differentiation of intestinal macrophages. IL-10 is crucial in inducing subtype of anti-inflammatory macrophages, maintaining the hyporesponsiveness to toll-like receptors (TLRs) stimulation, and promoting antigen-specific regulatory T (Treg) cells. CX3C-chemokine ligand 1 (CX_3_CL1), secreted by intestinal epithelial cells, plays a critical role in the differentiation and function of mouse intestinal macrophages. In addition, CX_3_CR1^+^ macrophages form transepithelial dendrites (TEDs) with the ability to capture potential pathogens and transfer antigens to dendritic cells. The transforming growth factor-β (TGF-β) is essential for the terminal differentiation of macrophages in intestinal mucosa, and genetic imprinting of intestinal macrophages in mice. Besides, efferocytosis produce the secretion of TGF-β by macrophages, restraining the production of pro-inflammatory mediators. Mature intestinal macrophages are accompanied with the upregulation of IL-10, TGF-β, IL-6, iNOS, and the ability of phagocytosis and TLR responsiveness. Created with BioRender.com.

It has been proposed that macrophage precursors might compete for a limited number of niches, and the balance between embryo-derived macrophages and bone-marrow derived monocytes is delicately orchestrated by niche accessibility, availability and precursor plasticity (Guilliams and Scott, 2017; Guilliams and Svedberg, 2021). The orchestration of the niche assures that monocytes can differentiate efficiently into macrophages based on tissue-specific microenvironments only when the niche is available (Guilliams and Scott, 2017). Niche imprints play a strategic role in dictating macrophage identity and capacity for self-maintenance rather than only their origin, as was described previously (Mass and Gentek, 2021; Viola and Boeckxstaens, 2021). When the body is in a steady state, embryonic macrophages continuously renew and maintain themselves in adult tissues (Louwe et al., 2021). In the case of infection and tissue damage causing large consumption of tissue-resident macrophages, blood circulating monocytes can be used for long-term reconstruction of tissue-resident macrophages (Louwe et al., 2021). Intestinal macrophages are replenished by blood-derived monocytes through a series of differential intermediates in a step-wise continuum way, which is a process taking around 5–6 days, and has become known as the monocyte waterfall (Bain et al., 2013; Bain et al., 2014). (Figure 1). Lymphocyte antigen 6C-high (Ly6C^hi^) CX3C-chemokine receptor 1- intermediate (CX3CR1^int^) major histocompatibility complex II (MHCII^−^) (P1) monocytes seem similar to their counterparts in blood in aspects of both phenotype and morphology (Schridde et al., 2017). As these cells enter the lamina propria, they first acquire the expression of MHCII (P2), and then downregulate Ly6C expression (P3), together with the other proteins of extravasation, and finally, upregulating the expression of CX_3_CR1, CD64, and F4/80, giving rise to mature macrophages (P4) (Bain et al., 2013; Bain et al., 2014) (Figure 1). Notably, more and more evidences suggest that there is an analogous waterfall in the human intestinal mucosa, with classical CD14^hi^CCR2^+^CD11c^hi^ monocytes gradually differentiating into CD14^low^CCR2^-^CD11c^low^ macrophages (Bain et al., 2013; Bernardo et al., 2018; Bujko et al., 2018). However, there are still many questions to be answered about this process. How do macrophages differentiate into specific compartments in the distinct micro-environment of the duodenum with powerful absorptive ability or the cecum with myriad microbes? Is there a role for tissue-resident macrophages in memorizing past inflammatory injury within the intestinal tissue? Based on the aforementioned findings and potential challenges, facilitating the restoration of inflammation-imprinted resident macrophages and their niche in homeostasis may bring about a promising therapeutic avenue to prevent secondary complications in these patients.

## 3 Function of Intestinal Macrophages in IBD

IBD is characterized by a destructive and relapsing inflammation of the gastrointestinal tract that harbors many macrophages that accumulate in the mucosa and are involved in the pathogenetic process. In IBD, the inflamed colonic mucosa can recruit a large number of macrophages, vigorously secreting IL-1, IL-6, TNF-α, IL-12, and IL-23, as well as producing reactive oxygen species (ROS), reactive nitrogen intermediates (RNIs), and proteases that could degrade the extracellular matrix. However, the characteristics of intestinal-resident macrophages in CD and UC are distinct with respect to distribution, ability to clear pathogens, and involvement in fibrosis. Macrophages mainly spread within the intestinal mucosa in UC, however, in CD, these cells also infiltrate in the muscular layer and mesenteric fat tissue (Mahida et al., 1989; Kredel et al., 2013). In the past, there was a simplified statement that CD has even been regarded as a primary immunodeficiency of macrophage (Casanova and Abel, 2009). Therefore, impaired cytokine secretion by macrophages was thought to be the reason for defective pathogenic clearance in CD (Smith et al., 2009). It is common to observe that intestinal macrophages contain *E. coli* in CD, but not in UC (Elliott et al., 2015). In contrary, bacterial pathogens were responded actively by macrophages in patients with UC (Rahman et al., 2010). As a consequence, the formation of granulomas is generally found in CD patients but not the UC patients, because of the failure to eradicate invading pathogens effectively (Cook and Dixon, 1973). Notably, the degree of fibrosis is more pronounced in CD than in UC (Yamagata et al., 2011). Excessive wound-healing processes involve various steps with macrophages, resulting in the development of fibrosis and fibrotic strictures subsequently. In fibrotic CD, several factors produced by macrophages promote the formation of fibrosis, such as MMP-2, IL-13, TNF-α, and transforming growth factor-β (TGF-β) (Fichtner-Feigl et al., 2006; Bailey et al., 2012).

### 3.1 Macrophage Subsets in IBD

Mature macrophage phenotypes and functions are highly plastic and heterogeneous in response to environmental cues (Mosser and Edwards, 2008). Conventionally, a dichotomic nomenclature describes activated macrophages as having two opposite states: classically activated (M1) and alternatively activated (M2) macrophages (Gordon, 2003; Aggarwal et al., 2014). The imbalance of M1/M2 phenotypes has been demonstrated to be involved in colitis exacerbation in the murine model of IBD (Zhu et al., 2014). Here, we summarize the stimulators of M1 and M2 macrophages, molecular marker expression, signaling pathways involved, major cytokines and chemokines secreted, as well as their functions in IBD.

**FIGURE 2:**
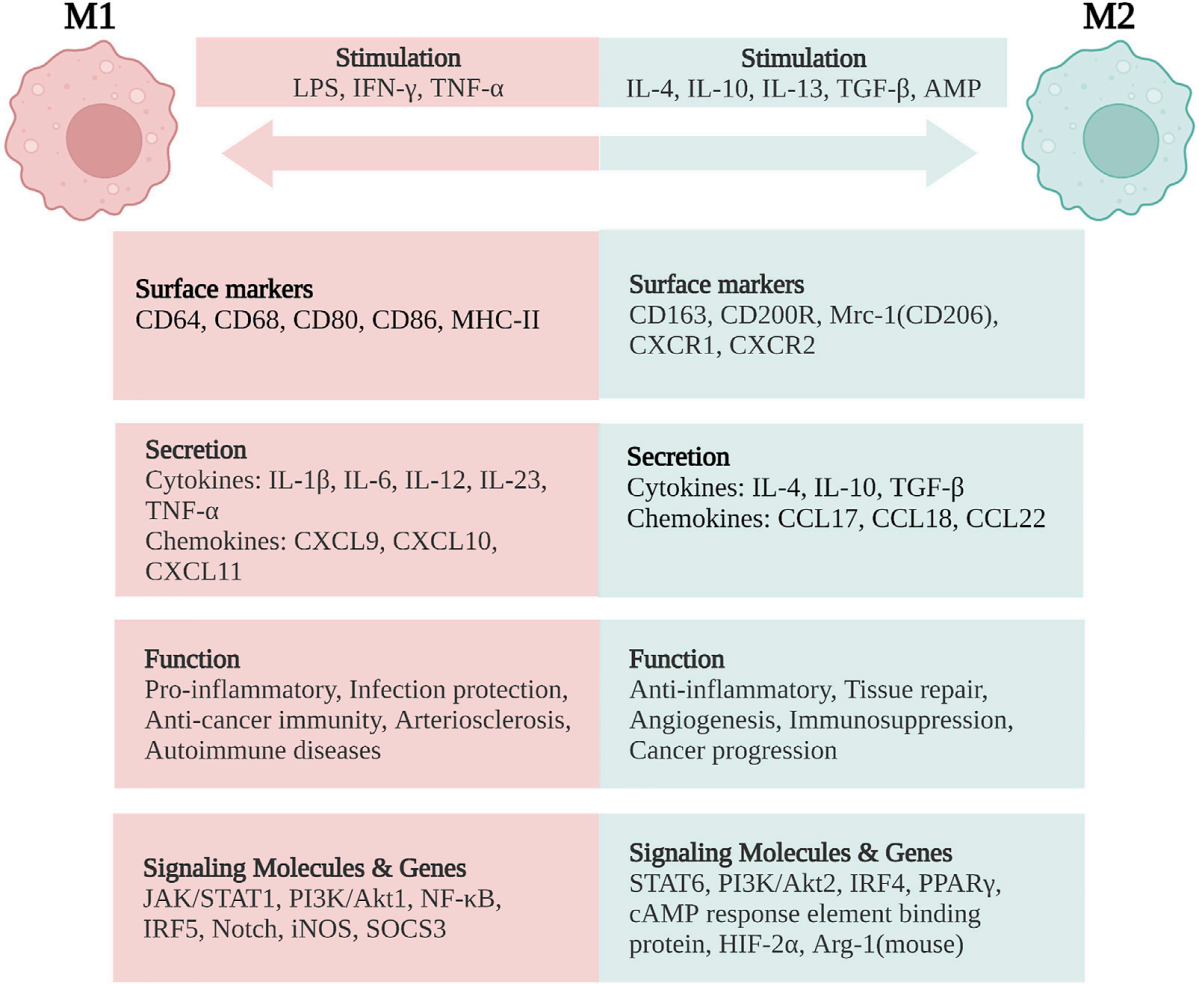
Macrophage polarization and phenotypes. M1 macrophages are stimulated by LPS, IFN-γ, and TNF-α, while M2 macrophages are stimulated by IL-4, IL-10, IL-13, TGF-β, and AMP. M1 macrophages are characterized by the functions of pro-inflammatory, infection protection, anti-cancer immunity, arteriosclerosis, and autoimmune diseases. M2 macrophages exert the functions of anti-inflammatory, tissue repair, angiogenesis, and immunosuppression, but are related with cancer progression. LPS, lipopolysaccharide; IFN, interferon; TNF, tumor necrosis factor; IL, interleukin; TGF, transforming growth factor; AMP, adenosine monophosphate; CD, cluster of differentiation; MHC, major histocompatibility complex; Mrc-1, mannose receptor C-type 1 (also known as CD206); CXCR, CXC chemokine receptor; CXCL, CXC chemokine ligand; CCL, CC chemokine ligand; JAK/STAT, Janus kinase/signal transducer and activator of transcription; PI3K/Akt, phosphatidylinositol-3-kinase/Akt; NF-κB, nuclear factor-κB; IRF, interferon regulatory factor; iNOS, inducible nitric oxide synthase; SOCS3, suppressor of cytokine signaling-3; PPARγ, peroxisome proliferator activated receptor-γ; HIF-2α, hypoxia-inducible factor-2α; Arg-1, arginase-1. Created with BioRender.com.

In general, classically activated (M1) macrophages are characterized by secreting cytokines such as IL-12, IL-23, IL-1β, TNF-α, ROS, and RNIs, inducing inflammation and clearing pathogens. In lamina propria of inflamed gut, pro-inflammatory M1 macrophages break down the tight junction proteins, damage the epithelial barrier, and induce epithelial cell apoptosis, leading to excessive inflammation (Cosín-Roger et al., 2013; Steinbach and Plevy, 2014; Lissner et al., 2015). In addition, the M1 subsets are also involved in the induction of T helper 1 cells (Th1) and Th17 responses. The transformation of macrophages into the M1 phenotype involves many key transcription factors, such as Janus kinase/signal transducer and transcription activator 1 (JAK/STAT1) (Hu et al., 2002; Gordon and Martinez, 2010), phosphoinositide 3-kinase (PI3K)/Akt1 (Lu et al., 2017; Vergadi et al., 2017), nuclear factor (NF)-κB (Oeckinghaus et al., 2011; Zhang et al., 2018), interferon regulatory factor 5 (IRF5) (Chistiakov et al., 2018), and Notch (Xu et al., 2012; Siebel and Lendahl, 2017). On the other hand, sustained M1 phenotype expression has been shown to aggravate the inflammatory response and ultimately cause tissue damage (Figure 2).

On the contrary, alternatively activated (M2) macrophages are usually characterized by upregulated factors such as IL-4, IL-10, mannose receptor C-type 1 (Mrc-1, also known as CD206), CD163, arginase-I, IL-1 receptor antagonist, and stabilin-1, which could prevent excessive inflammation, and promote tissue healing. M2 polarization is mainly induced by transcription factors including STAT6 (Ohmori and Hamilton, 1997; Martinez et al., 2009), PI3K/Akt2 (Lu et al., 2017; Vergadi et al., 2017), IRF4 (Satoh et al., 2010; Chistiakov et al., 2018), peroxisome proliferator-activated receptor (PPAR)γ (Bouhlel et al., 2007; Odegaard et al., 2007), and cyclic adenosine monophosphate (cAMP) response element binding protein (Ruffell et al., 2009; Luan et al., 2015). M2 macrophages promote resolving inflammation and remodeling tissue by producing several factors, especially IL-10. Some research has shown that the inhibition of M2 polarization or the synthesis of its cytokines may result in the exacerbation of colitis (Hunter et al., 2010; Zhu et al., 2014). M2 macrophages activate TGF-β by promoting Th2 responses to further promote fibrosis that is intimately involved in tissue remodeling (Murray and Wynn, 2011; Locati et al., 2013; Wynn and Vannella, 2016). In addition, M2 macrophages promote matrix metalloproteinases (MMPs) that control extracellular matrix (ECM) turnover to remove debris, apoptotic cells, and various ECM components, which could help to prevent tissue-damaging M1 macrophage responses (Murray and Wynn, 2011). Moreover, the expression of Arg1, Fizz1, and Ym1 has been shown to decrease inflammatory responses and promote tissue remodeling (Lawrence and Natoli, 2011; Kühl et al., 2015). According to different stimuli and transcriptional changes, the M2 macrophages could be subdivided into several subsets (M2a, M2b, M2c, and M2d subsets) with distinct pathophysiological characteristics (The details are summarized in Table 1) (Rőszer, 2015; Wang et al., 2019; Du et al., 2021). Based on these findings, the importance of macrophage polarization in the progression and prognosis of inflammation is becoming increasingly appreciated (Figure 2).

**TABLE 1:** Different subclasses of M2 macrophage.

Subclass	Stimuli	Cell markers	Chemokines	Cytokines and mediators	Functions
M2a	IL-4, IL-13	Mouse: Arg1, Fizz1, Chi3l3, CD206	CCL13, CCL17, CCL18, CCL22, CCL24	IL-10, Arg1, TGF-β, IGF, Fibronectin	Anti-inflammatory, wound healing, elimination of parasites
		Human: CD206, CD163^low^, IL-1R, CD14^low^			
M2b	LPS, IC, IL-1β	Mouse: CD80, CD86, IL-10^high^, IL- 12^low^, HLA-DR^low^, MHC II	CCL1	IL-1, IL-6, IL-10, TNF-α	Th2 activation, immunoregulation, promoting infection, tumor progression
		Human: CD14^high^, CD80^high^, CD200R^low^			
M2c	IL-10, Glucocorticoids， TGF-β	Mouse: Arg1, CD206	CCL16, CCL18, CXCL13	IL-10, TGF-β, Arg1, MerTK	Anti-inflammatory, tissue remodeling, phagocytosis, immunosuppression
		Human: CD86^low^, CD163^high^, CD206, TLR1, TLR8			
M2d	A2R ligands, LPS, IL-6	Mouse: IL-10^high^, IL-12^low^, TNF-α^low^, VEGF	CCL5, CXCL10, CXCL16	IL-10, VEGF, TGF-β	Anti-inflammatory, angiogenesis, tumor progression
		Human: VEGF, IL-10, TGF-β			

A2R, A2 adenosine receptor; Arg-1, arginase-1; CCL, chemokine (C-C motif) ligand; CD, cluster of differentiation; CXCL, chemokine (CX-C motif) ligand; Chi3l3, chitinase 3-like 3 (also known as Ym1); FIZZ1, found in inflammatory zone 1; IC, immune complex; IGF, insulin-like growth factor; LPS, lipopolysaccharide; MerTK, Mer receptor tyrosine kinase; TLR, Toll-like receptor; VEGF, vascular endothelial growth factor.

### 3.2 Intestinal Macrophages and Gut Microbiota in IBD

Gut commensal microbiota has continuous interaction with epithelial cells, which is essential in shaping the function of intestinal barrier structure (Hayes et al., 2018). The dysbiosis of gut microbiota has been considered as the key of IBD etiopathology (Gilbert et al., 2018). Pathogen/microbial-associated molecular patterns (PAMPs/MAMPs) are microbial signature molecules, which are recognized and attached by the innate intracellular receptors of macrophages. The macrophages present antigens to activate T cells, leading to the production of cytokines, chemokines, and antimicrobial peptides (AMPs) to maintain the integrity of intestinal barrier (Wells et al., 2011). Besides, the recruitment and differentiation of intestinal macrophage also need the crucial mediator of the microbiota, aside from the induction of local cytokines, growth factors, and chemokines (Fiocchi, 2008). A few of studies have shown in germ-free mice or in mice with depletion of microbiota with broad-spectrum antibiotics, could decrease the number of monocyte-derived or tissue-resident macrophages (Bain et al., 2014; Muller et al., 2014; Shaw et al., 2018). The metagenomic sequencing of microbial RNA has revealed that there are significant alterations in microbial composition, location and biodiversity between IBD patients and healthy individuals (Wallace et al., 2014).

For instance, the adherent invasive *E. coli* (AIEC) showed significant increased amount in IBD patients, and it is involved in IBD pathogenesis through evading the immune system of the host, linking to intestinal epithelial cells, and promoting excessive inflammation (Lloyd-Price et al., 2019). Besides, AIEC is associated with the ability to transverse the intestinal wall into the lamina propria, even survive and replicate within macrophages by escaping autophagy (Palmela et al., 2018). *Campylobacter* concisus (C. concisus) is a kind of adherent and invasive proteobacterium, participating in the pathogenesis of IBD (Underwood et al., 2016). Some C. concisus strains acquired zonula occludens toxin (zot) gene from a virus (prophage), damaging epithelial tight junctions, inducing macrophage production of undue pro-inflammatory cytokines, such as TNF-α, and ultimately causing the chronic relapse of IBD (Zhang et al., 2014). Akkermansia muciniphila (A. muciniphila) is a Gram-negative anaerobic bacterium, that revealed protective potential with reduced abundance both in CD and UC (Png et al., 2010). A. muciniphila or a specific outer membrane protein Amuc_1100 could improve colitis, with the decreased number of infiltrating macrophages and CD8^+^ cytotoxic T lymphocytes in the gut (Wang et al., 2020). In a word, it is recognized as a crucial challenge to reconstitute the relationship between the host and the microbiome to an effective symbiotic state.

### 3.3 Macrophage-Derived Exosome

Macrophage-derived exosome has been shown to play an important role in the pathogenesis of inflammatory exacerbation and resolution by interacting with intestinal epithelial cells (IECs) and other cells. Exosome-derived miR-21a-5p from M1 macrophages could be absorbed by IECs, correlated with downregulation of E-cadherin and activation of group 2 innate lymphoid cells, aggravating dextran sulfate sodium (DSS)-induced colitis in mice (Lu et al., 2021a). In addition, the M2 macrophage-derived exosome miR-590–3p targets large tumor suppressor kinase 1 and then activates Yes-associated protein/β-catenin transcription in IECs, hence reducing colonic inflammation, strengthening mucosal healing, and alleviating DSS-induced colitis in mice (Deng et al., 2021).

## 4 Regulation Mechanism of Macrophages in IBD

Generally speaking, as specialized phagocytic cells of the innate immune system, macrophages exhibit multiple functions such as the maintenance of systematic homeostasis, protective defense of the host, and regulation of inflammatory response, as well as re-establishing tissue barriers upon mucosal disruption (You et al., 2016; Dionne et al., 2017). In addition, intestinal macrophages have also been found to secrete various cytokines to maintain tissue homeostasis (Grainger et al., 2017). Here, we summarize the critical regulation mechanisms of macrophages in IBD.

**CSF-1.** Macrophage colony-stimulating factor 1 (CSF1) is the primary cytokine, which shows a critical role in the survival, proliferation and differentiation of intestinal macrophages. It has been demonstrated that the number of intestinal macrophages appears significant decrease in Csf1-null mice and these with the administration of anti-CSFR1 antibody (MacDonald et al., 2010). Besides, following anti-CSF1R treatment, the depletion of macrophage leads to restraining Paneth cell differentiation, reducing Lrg5^+^ stem cells, and ultimately impairing the development of IECs (Sehgal et al., 2018).

**IL-10/IL-10R Axis.** The IL-10/IL-10-receptor (IL-10R) axis is crucial in regulating phenotype of intestinal macrophages in both mice and humans. Lack of IL-10 signaling results in macrophage hyper-responsiveness to toll-like receptors (TLRs) stimulation, causing the development of spontaneous intestinal inflammation (Zigmond et al., 2014). Disrupted IL-10/IL-10R axis can affect several regulatory pathways involved in macrophage activation, such as STAT1, STAT3, nuclear factor-κB (NF-κB), and TREM-1, as well as increase chromatin accessibility to pro-inflammatory genes (Hirotani et al., 2005; Schenk et al., 2005; Simon et al., 2016). Besides, macrophage-derived IL-10 has been demonstrated that it is crucial for maintaining and promoting antigen-specific Treg cells in the intestinal mucosa in mice (Hadis et al., 2011).

**CX**_**3**_ **CL1-CX**_**3**_ **CR1.** The CX_3_CL1-CX_3_CR1 axis plays a critical role in the differentiation and function of mouse intestinal macrophages, considering that the high expression of CX_3_CR1 and their special localization near by the CX_3_CL1-positive intestinal epithelial cells. Lack of CX_3_CR1 expression of intestinal macrophages leads to the decrease of IL-10 and defect in promoting Treg cells in the lamina propria, which may exacerbate the DSS-induced colitis in mice (Hadis et al., 2011). In patients with CD, a missense mutation in the CX_3_CR1 gene was identified, which was related with an impaired antifungal ability and may lead to progression of extra-intestinal inflammatory diseases (Leonardi et al., 2018). In addition, CX_3_CR1^+^ macrophages can form transepithelial dendrites (TEDs) with the ability to capture soluble antigens or potential pathogens, and even migrate into the colonic lumen to execute the mission (Rescigno et al., 2001; Vallon-Eberhard et al., 2006).

**TGF-β.**The TGF-β/TGF-β-receptor (TGF-βR) axis is essential for the terminal differentiation of macrophages in intestinal mucosa, and genetic imprinting of intestinal macrophages in mice, including CX3CR1, IL-10, and αvβ5 integrin genes (Schridde et al., 2017). In line with this, the expression of the Runt-related transcription factor 3 (RUNX3), which is regulated by TGF-β, is a unique feature of intestinal macrophages (Lavin et al., 2014). In addition, macrophage turnover is regulated by the TGF-β/TGF-βR axis by controlling the expression of CCL8 (Schridde et al., 2017). Besides, efferocytosis produce the secretion of TGF-β by macrophages, restraining the production of pro-inflammatory mediators by a mechanism of autocrine/paracrine way involving TGF-β (Fadok et al., 1998).

## 5 Targeting Macrophages for IBD

**FIGURE 3:**
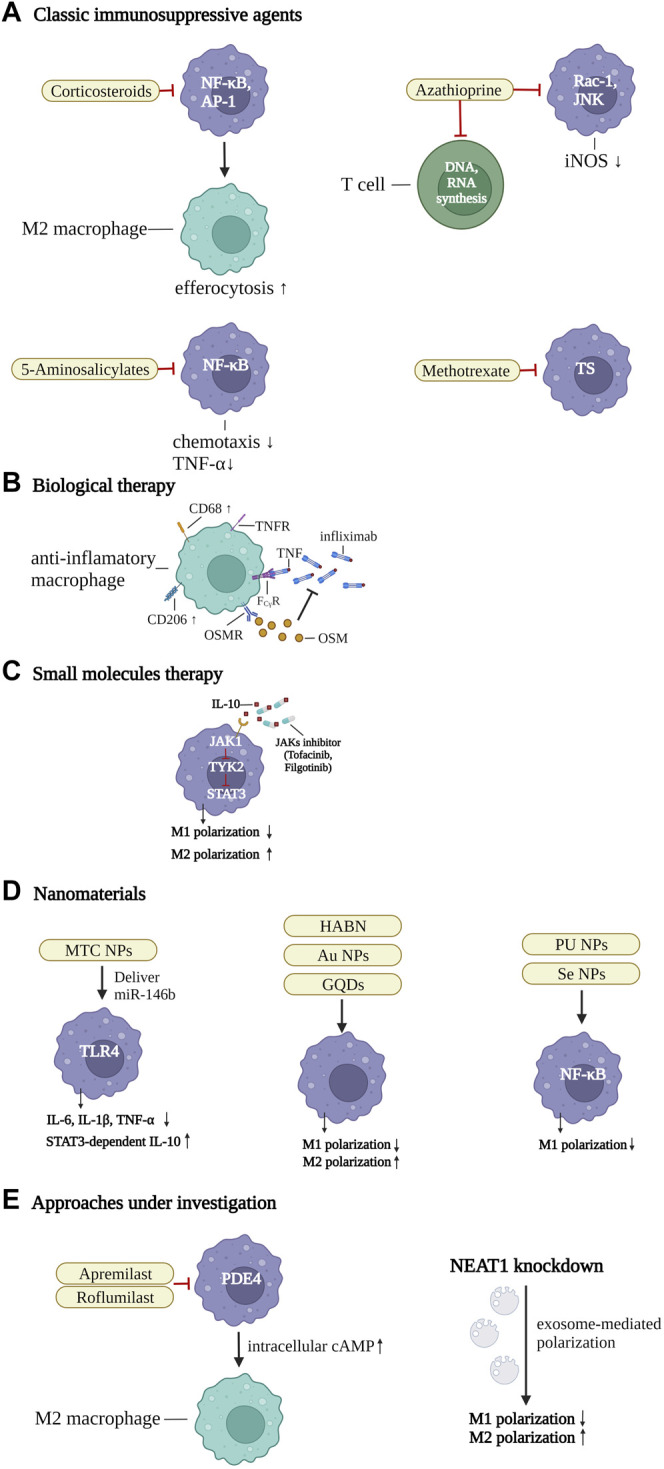
Therapeutic strategies of IBD by targeting the macrophages. **(A)** classic immunosuppressive agents. Corticosteroids inhibit nuclear factor-κB (NF-κB) and activator protein 1 (AP-1), thereby enhancing efferocytosis and inducing the differentiation of M2 macrophages. 5-Aminosalicylate suppresses NF-κB to downregulate TNF-α effects and control the chemotaxis of macrophages. Azathioprine not only restricts T-cell activation by abolishing DNA or RNA synthesis, but also suppresses Ras-related C3 botulinum toxin substrate 1 (Rac1) activity and the phosphorylation of JUN N-terminal kinase, and the expression of iNOS. Methotrexate regulates pro-inflammatory gene expression of macrophages, by controlling thymidylate synthase (TS), **(B)** biological therapy. Infliximab blocks TNF and TNF receptor (TNFR)-mediated inflammatory response in intestinal macrophages, by binding with Fcγ receptor (FcγR), and inducing CD68^+^CD206^+^ regulatory macrophages. The numbers of oncostatin M (OSM) and the receptor (OSMR-β) are associated with unresponsiveness to TNF-α blockers. **(C)** small molecules. JAK inhibitors inhibit the IL-10-JAK1-tyrosine kinase 2-STAT3 signaling pathway, which is crucial for maintaining mature macrophages in intestinal homeostasis. In addition, they can downregulate M1 and promote the differentiation of M2 macrophages. **(D)** nanomaterials. A series of macrophage targeting nanoparticles (NPs) could be synthesized to deliver specific pathway inhibitors or agonists to control inflammatory-related pathways and promote the polarization of anti-inflammatory macrophages, such as mannose-modified trimethyl chitosan (MTC) NPs, Hyaluronic acid-bilirubin nanomedicine (HABN), gold (Au) NPs, graphene quantum dots (GQDs), polyurethane (PU) NPs, and selenium (Se) NPs. **(E)** approaches under investigation. Apremilast and roflumilast are two new phosphodiesterase 4 (PDE4) blockers. The intracellular cAMP level was increased by inhibition of PDE4, thereby blocking inflammation-related factors and promoting pro-resolving macrophages. Inhibition of the Nuclear Enriched Abundant Transcript 1 (NEAT1), a new nuclear long non-coding RNA, can regulate the intestinal mucosal barrier and induce exosome-mediated differentiation of macrophages. Created with BioRender.com.

There is a huge driving force for exploring innovative treatment options in IBD, because existing therapeutic strategies remain ineffective in many patients (Papamichael et al., 2015). It has been shown that modulating the polarization of M2 macrophages or suppressing inflammation-related cytokines could be an innovative therapeutic strategy to regulate intestinal inflammation and restore tissue homeostasis (Na et al., 2019; Du et al., 2021). Modulating the phenotypes of macrophages by inhibiting the pro-inflammatory M1 subset and/or inducing the anti-inflammatory M2 subset, may ameliorate IBD effectively (Ma et al., 2019). It has also been shown that macrophage functions can be affected and modulated by classic IBD agents (such as, infliximab or mesalazine) in many ways, for instance, by downregulating inflammatory signaling pathways and/or skewing to the M2 subpopulation (Bantel et al., 2000; Vos et al., 2012). In the following paragraphs, the current treatment strategies involving macrophages and potential new options to enhance the accumulation of the pro-resolving subpopulation in IBD are summed up in detail (Figure 3).

### 5.1 Classic Immunosuppressive Agents

Conventional treatment strategies usually rely on modulating the systemic immune responses by mitigating progression of mucosal inflammation. These conventional strategies are mainly based on immunomodulatory medications such as corticosteroids, aminosalicylates, antibiotics, and thiopurines, as well as folic acid antagonists (methotrexate) (Sales-Campos et al., 2015; Chen et al., 2021).

As the central medication for acute exacerbations of IBD patients, both UC and CD, corticosteroids remain the cornerstone of therapeutic options at present (Mowat et al., 2011). Corticosteroids comprise a series of drugs that exerts anti-inflammatory effects by binding to particular cytosolic receptors and suppressing transcription factors, for instance, NF-κB and activator protein 1 (Oakley and Cidlowski, 2013). Moreover, the activation of immune cells and the production of adhesion molecules are suppressed by corticosteroids in inflamed sites (Sales-Campos et al., 2015; Chen et al., 2021). In addition, corticosteroids have several effects on macrophages, ranging from enhancing efferocytosis to inducing the polarization of the M2 subpopulation (Giles et al., 2001; Ehrchen et al., 2007). Nevertheless, if corticosteroids are administrated chronically, the occurrence of side effects (e.g., osteoporosis, metabolic disease, cardiovascular syndrome), along with the non-responsive subpopulation, will appear and remain a primary obstruction to sustained remission (Ford et al., 2011; Sales-Campos et al., 2015).

5-Aminosalicylates could control the chemotaxis of macrophages or promote the proliferation of intestinal mucosa cells by downregulating TNF-α effects as well as suppressing NF-κB signaling pathways directly, which play an integral role in pro-inflammatory macrophages in UC patients (Kaiser et al., 1999; Bantel et al., 2000). In addition, 5-aminosalicylates are often combined with corticosteroids in UC patients to reinforce the anti-inflammatory effects (Allgayer, 2003).

Azathioprine and methotrexate are the other two classic immunosuppressive drugs for IBD (Feagan et al., 2000). They not only restrict T-cell activation by abolishing DNA or RNA synthesis but suppress RAC1 activity *via* binding to 6-thioguanine triphosphates, resulting in anti-inflammatory functions in the mouse model by inhibiting the phosphorylation of JUN N-terminal kinase and the expression of iNOS. (Elion, 1989; Marinković et al., 2014). By controlling thymidylate synthase, methotrexate regulates pro-inflammatory gene expression of macrophages (Municio et al., 2016). Based on the aforementioned evidence, macrophage-modulating strategies play an important role in treating IBD patients.

### 5.2 Biological Therapy

TNF-α is a pro-inflammatory mediator that can be produced by macrophages and other cells including B cells, T cells, natural killer (NK) cells, dendritic cells (DCs), neutrophils, and epithelial cells (Kany et al., 2019). Macrophages are the major producers of TNF-α and interestingly are also highly responsive to TNF-α (Parameswaran and Patial, 2010).There is increasing evidence reinforcing that TNF-α plays a crucial role in the pathogenesis of IBD (Targan et al., 1997; D'Haens et al., 1999; Rutgeerts et al., 1999). The anti-TNF treatment remains a crucial strategy for IBD patients, given that it has the ability to promote the differentiation of regulatory macrophages (Vos et al., 2012).

TNF-α blockers have been used to prevent TNF-α-mediated intestinal mucosal damage and reduce or terminate the biological effects of TNF-α. Since the anti-TNF agent infliximab was approved by U.S. Food and Drug Administration in 1997, biologics have become the primary medications in the treatment of IBD patients (Stack et al., 1997; Targan et al., 1997). Thalidomide is a TNF-α inhibitor, which has been demonstrated to be effective for inducing remission in pediatric CD and adults with refractory CD (Sabate et al., 2002; Lazzerini et al., 2013). By blocking the production of interferon-regulatory factor-5 (IRF-5), thalidomide significantly inhibits the differentiation of M1 macrophage, with downregulated surfaced markers including CD86 and CCR7, as well as decreased pro-inflammatory cytokines including IL-12 and IFN-γ, that attenuates intestinal inflammation and facilitates mucosal healing (Lu et al., 2021b). Several new anti-TNFα agents, such as adalimumab, certolizumab pegol, and golimumab, have been applied in clinical research (Danese et al., 2015; Billmeier et al., 2016; Holleran et al., 2017; Chudy-Onwugaje et al., 2019; Na et al., 2019). The ENVISION I study is a phase Ⅲ double-blind randomized controlled clinical trial (RCT) investigating the clinical efficacy of adalimumab in pediatric patients with moderate-to-severe UC (Croft et al., 2021). After 8 weeks of treatment, the adalimumab group had significantly more frequent clinical remission than the placebo group (adalimumab group vs. placebo group; 60 vs. 19.8%, *p* = 0.0001) (Croft et al., 2021). Therefore, moderate-to-severe UC in children could be efficaciously and safely treated by adalimumab (Croft et al., 2021).

TNF-α inhibitors can specifically bind to receptors to form immune complexes (ICs) to neutralize TNF-α. Once bound to the antibody, TNF-α receptor activation is blocked, leading to decreased permeability of cytomembranes and paracellular tight junctions, as well as blocking the accumulation of inflammatory cells in the local intestinal mucosa (Baert et al., 1999; D'Haens et al., 1999; Rutgeerts et al., 1999; Koch and Nusrat, 2012; Vos et al., 2012; Olesen et al., 2016). In addition, by hindering lipid rafts, infliximab could rebuild equilibrium between the intestinal mucosal barrier and adherent invasive *E. coli* in CD (Yakymenko et al., 2018). In addition, anti-TNF medications could not only reduce the secretion of inflammatory cytokines and infiltration of T cells but also skew monocytes to differentiation into regulatory macrophages (CD68^+^CD206^+^), concurrently (Vos et al., 2012; Olesen et al., 2016). Compared to anti-TNF-α monotherapy, TNF-α inhibitors combined with thiopurines have been demonstrated to promote the differentiation of regulatory macrophages and enhance immunosuppressive efficacy (Vos et al., 2012). However, a majority of IBD patients are intolerant to the therapeutic regimen of anti-TNFα combined with thiopurine, which makes the strategy difficult to carry out (Jharap et al., 2010). Hence, ongoing studies should focus on finding novel alternatives for anti-TNFα combined treatment.

In addition, anti-TNF agents can induce alternative macrophage polarization *via* Fcγ receptor ligation (Lucas et al., 2005; Colombel et al., 2010; Pander et al., 2011; Vos et al., 2011; Vos et al., 2012) and increase IL-10 production of macrophages (Pander et al., 2011; Vos et al., 2011; Levin et al., 2016; Bloemendaal et al., 2017; Koelink et al., 2020). After TNF-α blocker treatment, there was obvious differentiation of the M2 subpopulation in patients with mucosal healing, whereas those without mucosal healing had no such induction (Vos et al., 2012). However, the anti-inflammatory subpopulation cannot be induced by biologics without the Fc fragment, for example, certolizumab pegol, indicating that the research on novel antibody-based strategies may be a broad avenue for IBD therapy (Boyer et al., 2016).

Moreover, the expression of oncostatin M (OSM) is elevated in IBD patients and is associated with unresponsiveness to TNF-α blockers (West et al., 2017). As a member of the IL-6 cytokine family, OSM comprises IL-11, IL-31, and leukemia inhibitory factor (Rose and Bruce, 1991). When the OSM gene was deleted or blocked, experimental colitis was significantly alleviated in animal models with resistance to TNF-α blockers (West et al., 2017). It's worth noting that OSM aggravates the inflammatory response and damages the intestinal mucosal barrier *via* initiating the expression of cytokines, chemokines, and adhesion factors, which produce large numbers of the receptor (OSMR-β) (Pothoven and Schleimer, 2017; West et al., 2017). Thus, OSM emerges as a novel therapeutic target for IBD patients, especially for those who are non-responsive to TNF-α inhibitors, and it may also promote remodeling of the intestinal tissue.

Even if some protocols of IBD therapy use biologics in an early top-down way in high-risk CD patients, the ineffectiveness of the therapy is still an intractable problem that drives patients to increase dosage of medications or try other therapeutic regimens (Berg et al., 2019; Maronek et al., 2020; Roda et al., 2020). As mentioned above, TNF-α blockers can exert anti-inflammatory effects and remodel the intestinal mucosal barrier by inducing the polarization of M2 macrophages, but further research is needed, especially in unresponsive patients.

### 5.3 Small Molecules

Janus kinases (JAKs) are non-receptor tyrosine kinases that comprise JAK1, JAK2, JAK3, and tyrosine kinase 2 (TYK2) (Darnell et al., 1994). Once the JAK cytokines bond to the cell-membrane receptors, the signaling transducers with activators of transcription (STATs) are activated, leading to STAT homo-dimerization and activate downstream transcription (Boland and Vermeire, 2017). Cytokines essential for intestinal homeostasis and those that are well-described mediators of pathological responses in IBD are all dependent on JAK/STAT-mediated signaling (Villarino et al., 2017; Paramsothy et al., 2018; Salas et al., 2020). Inhibition of the JAK/STAT pathway results in a decrease in several pro-inflammatory factors, for instance, IL-6, IL-12, and IL-23 (Schwartz et al., 2017). Therefore, JAK inhibitors may represent an emerging option for treating IBD (Na et al., 2019; Salas et al., 2020).

Tofacitinib, an oral pan-JAK blocker, has recently been approved for oral treatment of moderate-to-severe active UC (Kremer et al., 2012; Machado et al., 2018; Tripathi and Feuerstein, 2019; Rogler, 2020). It inhibits JAK1 and JAK3 preferentially, parts of the tyrosine kinase family, modulates cytokine secretion, and controls immunomodulation of IBD (Shivaji et al., 2019; Sandborn et al., 2021). Three phase Ⅲ trials, with 1,732 patients in total, showed that tofacitinib was more effective efficacy than placebo in inducing and maintaining remission in patients with moderately to severely active UC (Sandborn et al., 2017). However, another two phase Ⅱ double-blind RCT in CD patients, tofacitinib did not show significant results compared with placebo (Sandborn et al., 2014; Panés et al., 2017). Patients on high steroid dosages, some distinct disease characteristics, and details in research design (e.g., no endoscopic confirmation in center) may explain why the lack of efficacy was observed in CD, as opposed to UC.

Filgotinib, a selective JAK1 inhibitor, has emerged as a potential reliable therapy strategy in IBD patients. A phase Ⅱ double-blind RCT, the FITZROY study, included patients with moderate-to-severe CD (Vermeire et al., 2017). After 10 weeks of treatment, patients who were given filgotinib (200 mg, once daily) showed significantly more frequent clinical remission than patients taking placebo (filgotinib group vs. placebo group; 47 vs. 23%, *p* = 0.0077) (Vermeire et al., 2017). Another phase Ⅱb/Ⅲ double-blind RCT in patients with moderate-to-severe UC, the SELECTION study, showed that filgotinib (200 mg, once daily) was well tolerated and effective in induction and maintenance of clinical remission compared with placebo (Feagan et al., 2021). A number of studies focusing on the efficacy of this JAK inhibitor in CD patients are under way. The Divergence 2 study (NCT03077412), focusing on analyzing the efficacy of filgotinib in the treatment of perianal fistula in CD, has been completed but no results have yet been reported. Another phase Ⅲ double-blind RCT, The DIVERSITY1 (NCT02914561), will evaluate the effectiveness and safety of filgotinib in induction and maintenance therapy in patients with moderate-to-severe CD groups, who are biologic-naive and biologic-experienced. In addition, the DIVERSITYLTE trial (NCT02914600), aimed at evaluating the long-term safety in CD patients, is the extension of the DIVERSITY1 study.

JAK blockers could modulate functions of T cells, but also inhibit the IL-10-JAK1-tyrosine kinase 2-STAT3 signaling pathway, which is crucial for maintaining mature macrophages in intestinal homeostasis (Pattison et al., 2012). Interestingly, recent studies have shown that tofacitinib may affect the phenotype of macrophages, which was not investigated in previous related studies (Shiratori et al., 2018; De Vries et al., 2019). Studies have found that tofacitinib can downregulate M1 and promote M2 polarization (CD206, CD163, and IL-10) in murine bone marrow-derived or blood-derived macrophages (Shiratori et al., 2018; De Vries et al., 2019). In UC patients, tofacitinib significantly inhibited the M1 pro-inflammatory pathway, resulting in reduced expression of inflammatory factors; the M2 anti-inflammatory pathway was not affected, such as IL-10 secretion and expression of CD39 and CD206, even though the M2 markers were not upregulated (Cordes et al., 2020).

Targeting multiple pro-inflammatory cytokines, especially the JAK-STAT signaling pathway, may play an important role in novel IBD strategies, especially when patients are unresponsive to current regimens. The therapeutic mechanism of macrophage involvement in JAK blockers, especially in the process of resolving intestinal inflammation, needs to be confirmed in further research. In addition, side effects of JAK blockers are still of concern, such as the threat of thromboembolic events, opportunistic infections, or unknown long-term effects. The potential value of JAK blockers in the treatment of IBD needs to be further evaluated in high-quality and long-term RCTs, based on the risk-benefit ratio criterion.

### 5.4 Nanomaterials

Traditional medical regimens mainly rely on immunosuppressive agents, which could result in off-target systemic adverse effects and toxic reactions. As an emerging and prospective strategy, nanotechnology could be used for targeted delivery of medications to the inflamed lesions, enhancing local concentration as well as minimizing systematic adverse effects (Hur et al., 2012; Beloqui et al., 2016; Sohail et al., 2019). It has been shown that macrophage-targeted drug delivery systems can improve drug-delivery efficiency and decrease side effects by making full use of the phagocytic function of macrophages. This strategy has been widely explored in rheumatoid arthritis (Barrera et al., 2000), adiposis (Bu et al., 2013), and malignant tumors such as hepatocellular carcinoma and melanoma (Piaggio et al., 2016).

Intriguingly, macrophage-targeting nanoparticles have been synthesized to deliver inhibitors or agonists of inflammation-related pathways to modulate transition of macrophage phenotype (Singh et al., 2017). Nanomaterials-based therapeutics have shown great promise in the therapy of IBD, because of their useful properties in food and medicine (Cui et al., 2020). For example, miR-146b can be specifically delivered to intestinal macrophages by mannose-modified trimethyl chitosan (MTC)-conjugated nanoparticles (NPs), resulting in remarkable restoration of mucosal barrier function in the mice models (Deng et al., 2019). MTC-NPs can dramatically inhibit the activation of the M1 subset and induce the M2 phenotype by suppressing the TLR4 signaling pathway, thereby inhibiting the secretion of IL-6, IL-1β, and TNF-α (Deng et al., 2019). Moreover, by producing STAT3-dependent IL-10, the proliferation of co-cultured colonic epithelial cells was significantly increased (Deng et al., 2019). In addition, surface-functionalized polyurethane (PU) NPs showed remarkable immunosuppression of THP-1 macrophages, suppressing the secretion of inflammation-related cytokines in phenotypic M1, controlling the NF-κB signaling pathways, and ultimately modulating the differentiation of macrophages (Huang et al., 2018; Nakkala et al., 2021). Additionally, oral nanomedicine delivery systems for treating IBD have great promise, because of their expandability, biocompatibility, and potential targeted efficacy. Hyaluronic acid-bilirubin nanomedicine (HABN) is responsive to ROS, accumulating in inflammatory mucosa and remodeling the colonic tissue in experimental colitis. Importantly, HABN showed significant treatment efficacy *via* differentiating pro-inflammatory M1-like subtypes into the M2-like subtypes and elevating the expression of anti-inflammatory phenotypes such as CD3^+^CD4^+^Foxp3^+^ Tregs and CD11b^+^Ly6C^−^Ly6G^−^MHCII^+^ tissue-resident macrophages (Lee Y. et al., 2020; Xiao et al., 2021).

In recent years, metal-based engineered nanomaterials have shown advantageous properties in medicine. A classical case is that gold NPs (AuNPs) can induce M2 macrophage polarization, leading to inhibition of pro-inflammatory cytokines and increasing IL-10, Arg1, and PPARγ with decreased iNOS (Taratummarat et al., 2018). In addition, lines of evidence have demonstrated that nonmetal-based nanomaterials are capable of regulating macrophage phenotype. For example, graphene quantum dots (GQDs) could effectively alleviate intestinal inflammation and prevent tissue degeneration, *via* suppressing TH1/TH17 polarization (Lee B.-C. et al., 2020). Furthermore, GQDs can not only switch the differentiation of macrophages from pro-inflammatory M1 to anti-inflammatory M2 but also induce infiltration of intestinal Tregs (Lee B.-C. et al., 2020). A similar study also investigated the effect of nonmetal-based nanomaterials on macrophage polarization by using selenium (Se) (Zhu et al., 2017). Se is an indispensable nutrient and its deceased level may be associated with the exacerbation of IBD (Castro Aguilar-Tablada et al., 2016; Zhu et al., 2017; Ala and Kheyri, 2021). SeNPs have anti-inflammatory activity and low toxicity. Mechanistically, by inhibiting the nuclear translocation of NF-κB, SeNPs strongly suppressed M1 subsets, leading to alleviation of DSS-induced acute colitis in mice models (Zhu et al., 2017). Nanomaterials could be a promising therapeutic strategy for IBD with great potential in the near future.

### 5.5 Approaches Under Investigation

Inflammatory events are like a complex cascade, modulated by various cytokines and chemokines. Enhancing the pro-resolving phenotype by pharmacological modulation of the Inflammation-related molecules is of high importance to modulate macrophages in IBD. cAMP plays a key role in the modulation of inflammation-related process. Phosphodiesterase-4 (PDE4) is a critical enzyme, regulating intracellular signaling by controlling cAMP, emerging as a novel therapeutic target. Apremilast and roflumilast are two new PDE4 blockers, introduced as new regulators of intracellular signaling pathways in treating IBD patients (Spadaccini et al., 2017; Sabino et al., 2019). The intracellular cAMP level was upregulated by inhibition of PDE4, thereby blocking inflammation-related factors and increasing secretion of anti-inflammatory proteins (Mazur et al., 2015; Spadaccini et al., 2017). Moreover, a double-blind phase II trial of apremilast in patients with active UC showed significant remission of clinical symptoms, changes in endoscopic features, and decrease in markers of inflammation than in the placebo group (Danese et al., 2020).

Nuclear Enriched Abundant Transcript 1 (NEAT1), a new nuclear long non-coding RNA, anchors on particular nuclear structures and participates in various aspects of the immune process (Liu et al., 2018). It has been demonstrated that suppression of the NEAT1 can regulate the intestinal mucosal barrier and induce exosome-mediated differentiation of macrophages, thereby inhibiting the inflammatory response in IBD (Liu et al., 2018).

Targeting PDE4 or NEAT1 might represent a novel strategy in the future, but more high-quality clinical trials are still required to be carried out.

## Summary and Prospect

IBD is a prevalent gastrointestinal inflammatory disease, characterized by chronic and excessive inflammation, alternates states of relapse and remission, and needs for lifelong medical therapy (Hazel and O'Connor, 2020). Therefore, new strategies to prevent inflammatory relapse, tissular damage, and non-responsiveness to medications, are urgently needed.

The infiltration and activation of macrophages can phagocytize pathogens, as well as produce various cytokines under certain circumstances, and cooperate with distinct immune cells in many aspects of the pathogenesis of IBD. Disequilibrium of macrophage polarization results in the exacerbation of IBD, and the production of particular cytokines and/or chemokines relies on the ratio of the pro-inflammatory M1 and anti-inflammatory M2 subsets. Therefore, targeted therapy of macrophages is a novel option to modulate the immune microenvironment and remodel intestinal tissue.

In recent years, remarkable advances have been achieved in the understanding of intestinal macrophage immunobiology. The advancement of single-cell sequencing technologies and fate-mapping approaches has helped to confirm new subsets and distinct transcriptional profiles of intestinal macrophages (Ginhoux et al., 2010; Yona et al., 2013; Bian et al., 2020). Emerging evidence strongly points to an accumulation in the pro-inflammatory monocyte/macrophage in the chronic relapse and remission in patients with IBD. Therefore, it is very crucial to elucidate the mechanisms of new molecules and signaling pathways that participate in the polarization and increase of pro-resolving macrophages. However, many aspects of intestinal macrophages are still under-investigated, including mechanisms underlying macrophage polarization and heterogeneity, the signaling pathways of macrophage functions, environmental cues regulating phenotypes, and interactions during the process of IBD. For instance, it will be crucial to explore the characteristics of distinct subpopulations of macrophages, particularly the tissue-resident macrophages deriving from the embryo. This will facilitate finding out differences among distinct macrophages, expanding our comprehension of the functions of macrophages. In addition, the identification of microenvironmental factors that modulate phenotypes and functions of intestinal macrophage has helped us to understand their fundamental role in homeostasis and autoimmune disease. Moreover, research on cross-talk of intestinal macrophages, gut microflora, and metabolites will be conducive to decreasing chronic inflammation. It is also crucial that future investigations probe the synergistic mechanisms of cross-talk between macrophages and other immune cells in IBD.

Nanomedicine-based approaches have emerged as novel and targeted therapeutic strategies for IBD, by improving delivery efficiency to the intestinal inflammatory sites (Hua et al., 2015; Giron et al., 2019). Even though potential application of NPs has been broadly investigated in mammalian models *in vitro* or *in vivo*, human studies are still insufficient, and some findings in humans are distinct from the ones observed in murine models to some degree (Nunes et al., 2019). In addition, since the studies of toxicity and interaction of NPs have been mainly observed in animal models, efficacy in human intestinal cells is not fully applicable. The complexity of NP design and suitable methods for administration are two main difficulties with NPs, which impede the manufacturing process. Up to now, there has been no optimal size range of NPs determined to enable effective delivery to the cells in inflamed sites. In addition, the fate of the NPs after the body’s uptake is still unknown, because the majority of the studies have focused on delivery efficiency but not safety of the nanoparticle. Safety issues should be addressed first before moving toward clinical studies. Moreover, evaluation of the effectiveness of drug delivery is also critical. Using non-invasive methods, such as positron emission tomography, to image and trace macrophages in pharmacological responses is a potential option to evaluate the safety and delivery efficacy (Weissleder et al., 2014; Kim et al., 2018; Heo et al., 2019).

Many investigations have explored inventing novel medications and repurposing classical drugs, as well as searching for alternative strategies or improving the existing ones in the effort to eradicate IBD. More efforts are needed to create new strategies for treatment options, incorporating existing therapies, and adjusting the therapeutic schedules to better fit individualized treatment. Hence, future research needs to focus on translating the results from pre-clinical studies to be further explored in clinical trials, ultimately benefiting patients with IBD.
